# Impact of breast density on the efficacy of radiofrequency ablation in early-stage breast cancer

**DOI:** 10.1007/s12282-025-01775-7

**Published:** 2025-09-22

**Authors:** Manabu Futamura, Yasuko Nagao, Yukiko Takai, Yoshimi Niwa, Akira Nakakami, Mai Okawa, Yoshihisa Tokumaru, Junichi Mase, Ryutaro Mori, Daichi Watanabe, Takayuki Kinoshita, Nobuhisa Matsuhashi

**Affiliations:** 1 Department of Breast Surgery, Gifu University Hospital, 1-1 Yanagido, Gifu, 501-1194 Japan; 2 Department of Breast Surgery, Gifu Prefectural General Medical Center, Gifu, 500-8717 Japan; 3 Department of Radiology, Gifu University Hospital, Gifu, 501-1194 Japan; 4 Department of Breast Surgery, Central Japan International Medical Center, Minokamo, 505-8510 Japan; 5 Department of Medical Informatics, Gifu University Hospital, Gifu, 501-1194 Japan; 6 Innovative and Clinical Research Promotion Center, Gifu University Hospital, Gifu, 501-1194 Japan; 7 Department of Pharmacy, Gifu University Hospital, Gifu, 501-1194 Japan; 8 Department of Breast Surgery, NHO Tokyo Medical Center, Tokyo, 152-8902 Japan; 9 Department of Gastroenterological Surgery, Gifu University Hospital, Gifu, 501-1194 Japan

**Keywords:** Radiofrequency ablation (RFA), Early breast cancer, Breast density, Thermal conductivity

## Abstract

**Background:**

Radiofrequency ablation (RFA) is a minimally invasive technique employed in the management of small breast tumors. RFA involves the delivery of a high-frequency current through a needle electrode under ultrasound guidance. In Japan, RFA has been covered by insurance since 2023 as a localized treatment option for early-stage breast cancers.

**Methods:**

We retrospectively analyzed the data of patients who underwent RFA at our institution between February 2016 and March 2025. Breast density was classified into four categories based on the Breast Imaging Reporting and Data System. Associations between breast density and RFA parameters, including ablation temperature, time, and impedance, were evaluated.

**Results:**

A total of 50 breasts in 49 female patients were treated with RFA. The mean peak ablation temperature recorded after the cooling break was 81.0 ± 8.0 °C. Increased breast density was significantly associated with high temperatures. The mean ablation time until break was 446 ± 139 s, indicating a trend toward prolonged durations in cases involving denser breast tissue. The mean initial and final impedance values were 208 ± 72.3 Ω and 161 ± 66.5 Ω, respectively. Fat-rich breasts exhibited significantly higher impedance values at both time points.

**Conclusion:**

Fatty breast tissue is associated with higher impedance, lower peak temperatures, and shorter ablation times, potentially resulting in insufficient ablation. Breast density should be considered when planning RFA to ensure optimal treatment efficacy.

**Supplementary Information:**

The online version contains supplementary material available at 10.1007/s12282-025-01775-7.

## Introduction

Multidisciplinary therapy is essential for treating breast cancer, integrating local modalities such as surgery and radiation therapy with systemic treatments including hormone therapy, chemotherapy, molecular targeted therapy, and immunotherapy. Surgical treatment plays a pivotal role in achieving local control, with the standard approach evolving from radical mastectomy (Halsted procedure) to more conservative techniques, including modified radical mastectomy (Auchincloss method) and breast-conserving therapy involving partial mastectomy [1, 2]. The primary goal of breast oncologists is to achieve curative outcomes while balancing oncological safety with aesthetic preservation. With advancements in neoadjuvant chemotherapy, particularly for breast cancer subtypes such as triple-negative and human epidermal growth factor receptor 2 (HER2)-positive tumors, more than 60% of patients can now achieve a pathological complete response [3–6]. Consequently, several clinical trials are underway to evaluate the feasibility of omitting surgery in selected cases [7, 8]. Radiofrequency ablation (RFA) has emerged as a novel local treatment option for early-stage breast cancers in Japan.

RFA involves the percutaneous insertion of a needle electrode into small breast tumors under ultrasound (US) guidance, delivering high-frequency alternating current to generate Joule heat and ablate the tumor tissue [9]. Originally developed as a local treatment for hepatocellular carcinoma, RFA was first reported for use in breast cancer by Jeffery et al. in 1999 [10]. Adipose tissue has an electrical conductivity of less than 0.1 S/m, which is significantly lower than that of muscle tissue, clearly indicating its strong electrical insulation properties [11]. Therefore, breast tissue with high fat content can be considered to possess strong electrical insulating properties. Furthermore, since the background tissue differs from that of solid organs such as the liver or bone, various challenges must be addressed to ensure the efficacy and safety of RFA when applied to the breast [12]. Phase I and II feasibility studies, primarily conducted in Japan and Europe, have demonstrated the safety and clinical efficacy of this treatment [13–22]. In Japan, the RAFAELO study, the world’s first phase III trial for RFA in breast cancer, was launched in 2017, targeting patients with solitary tumors measuring ≤ 1.5 cm. The five-year outcomes were favorable, with an ipsilateral breast tumor recurrence-free survival rate of 98.6% and a local recurrence rate of only 0.57%, comparable to those observed following traditional breast-conserving surgery [23]. Based on these findings, regulatory approval was granted in July 2023 for the Cool-tip™ RFA system E series (cool-tip) (Medtronic plc, MN, USA) for use in RFA of early-stage breast cancer [24]. Moreover, insurance coverage for RFA was introduced in December 2023. To examine the efficacy of RFA in early-stage breast cancer, we initially conducted feasibility testing through trial excision. Subsequently, we participated in the RAFAELO and PO-RAFAELO studies conducted under patient-requested therapy [25] and RFA is not offered as part of routine insurance-covered treatment. However, the current knowledge regarding RFA remains limited. Therefore, in this study, we retrospectively and prospectively collected the clinical data from all patients who underwent RFA at our institution to examine the relationship between RFA treatment outcomes and mammographic breast density.

## Materials and methods

### Study population and design

In this single-institution observational study, we examined the relationship between background breast density and RFA parameters, including ablation temperature, time, and impedance, in patients who underwent the procedure at the Department of Breast Surgery, Gifu University Hospital, between February 1, 2016, and March 31, 2025. Clinical data were retrospectively collected from the electronic medical records. This study was approved by the Central Ethics Committee of Gifu University (Approval No. 2024-328).

### Patient selection

The patients were selected according to the RAFAELO study protocol. Eligible cases included patients with a solitary tumor ≤ 1.5 cm in maximum diameter, as assessed by both US and magnetic resonance imaging (MRI). All patients treated with RFA, including those enrolled in the feasibility study involving trial excision, the RAFAELO study, the PO-RAFAELO study, as well as those treated under the national insurance system, were included in the analysis.

### RFA procedure

The cool-tip device was utilized in all the procedures. Ablation was performed in accordance with established guidelines for the appropriate use of RFA in early-stage breast cancer. A radiofrequency electrode needle was inserted into the tumor under general anesthesia with US guidance. After the cool-tip electrode was punctured into the center of the tumor, the output power was initiated at 5 watts (W), increased to 10 W after 1 min, and subsequently raised by increments of 5 or 10 W every minute thereafter. No predefined upper limit was set for the output power. If the power limit of the device was reached, ablation was continued at the maximum output level. As the ablation progressed, the electrical impedance increased. When impedance exceeded a predetermined threshold, the system automatically ceased output, a phenomenon referred to as a “break” or “roll-off.” Following the first break, the pump was stopped, and the temperature at the needle tip was assessed. If the ablation temperature was below 70℃, the procedure was deemed insufficient, and an additional ablation was performed at the same site. If multiple additional applications still failed to reach 70 °C, a decision to continue or discontinue the procedure was made based on post-ablation imaging findings. To prevent thermal injury, a 5% glucose solution (totally 10–20 mL) was injected in both the retromammary space and subcutaneous tissue [15, 26].

### Breast density evaluation

Breast density was assessed using mammograms obtained before treatment. According to the fifth edition of the Breast Imaging Reporting and Data System (BI-RADS), the breast composition was classified qualitatively based on the relative proportions of fatty and fibroglandular tissues. This assessment provides critical information for evaluating breast density, which may influence the sensitivity of mammographic imaging and the risk of breast cancer worldwide [27]. Three board-certified radiologists independently evaluated the images using the BI-RADS classification system, which comprises four categories: Category A, almost entirely fatty; Category B, scattered areas of fibroglandular density; Category C, heterogeneously dense; and Category D, extremely dense. Two radiologists performed the initial assessment; in cases of discrepancy, a final determination was made by a third radiologist, who served as the most experienced supervising physician.

### Statistical analysis

All statistical analyses were performed using R version 4.4.2 (R Foundation for Statistical Computing, Vienna, Austria). For the outcome variables, ablation temperature, ablation time, initial impedance, and final impedance, the mean values and corresponding 95% confidence intervals (CI) were calculated for each breast density category.

An unpaired Student’s t-test was utilized for pairwise comparisons of these outcomes between breast density categories. Pearson’s correlation coefficient was employed to assess the relationship between tumor size and ablation parameters.

A post-hoc power analysis was performed based on the observed effect sizes, which were calculated using Cohen's d. Statistical significance was set at *p* < 0.05.

## Results

**Fig. 1:**
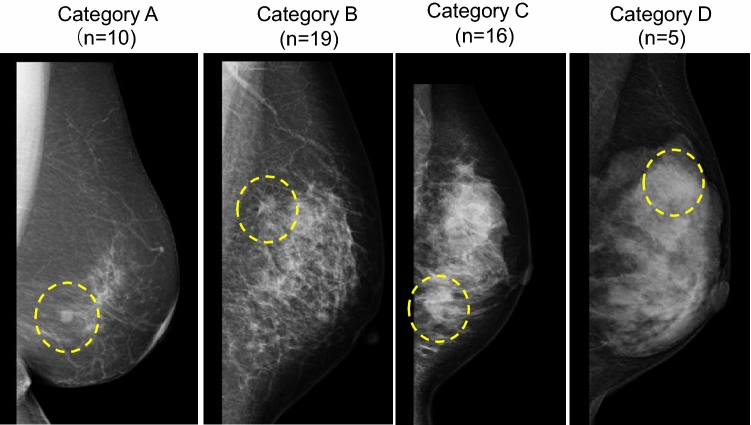
Breast density evaluated by mammography. Breast density was classified into four groups based on the Breast Imaging Reporting and Data System: Category A, almost entirely fatty (*n* = 10); Category B, scattered areas of fibroglandular density (*n* = 19); Category C, heterogeneously dense (*n* = 16); and Category D, extremely dense (*n* = 5). The area outlined by the dotted yellow line indicates early-stage breast cancer

RFA was performed on 50 breasts in 49 female patients. The mean age was 58.5 years, with the average body mass index (BMI) of 23.7 kg/m^2^. Among the 50 treated breasts, five were included in the feasibility study with trial excision, one in the RAFAELO study, 21 in the PO-RAFAELO study, and 23 were treated under national insurance coverage. Regarding the histological classification, 41 patients were diagnosed with invasive ductal carcinoma (IDC), while nine had ductal carcinoma in situ. Among the IDC subtypes, 39 were luminal A, whereas two were luminal/HER2. Breast density was categorized as A (*n* = 10), B (*n* = 19), C (*n* = 16), or D (*n* = 5) (Table 1 and Fig. 1).

**Table 1:** Patient characteristics

Gender	Female: 49
Age (yo)	58.5 ± 10.7(40–81)
BW (kg)	58.9 ± 8.5(42–78.8)
BMI	23.7 ± 3.4 (18–34.9)
Uni/Bilateral	Unilateral: 48, Bilateral: 1
	Right: 30, Left: 20
Tumor location	upper inner: 13,
	lower inner: 1,
	upper outer: 25,
	lower outer: 10
	under nipple: 1
Histology	IDC: 41, DCIS: 9
Subtype (only IDC)	Luminal A: 39, Luminal/HER2: 2
RFA	Feasibility study with trial excision: 5
	RAFAELO study: 1
	PO-RAFAELO study: 21
	National insurance coverage: 23
Breast density^a^	Category A: 10
	Category B: 19
	Category C: 16
	Category D: 5

BW: Body weight, BMI: Body mass index, IDC: Invasive ductal carcinoma; DCIS: Dactal carcinoma in situ

Data are shown as mean ± standard deviation (SD), with the range in parentheses

^a^Evaluated by the Breast Imaging Reporting and Data System

**Fig. 2:**
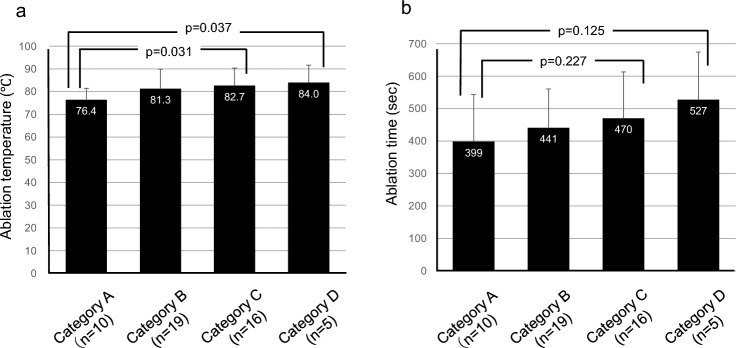
Relationship between breast density and peak ablation temperature and duration. **a** Mean ablation temperatures (℃) for each breast density category. **b** Mean ablation time (s). White numerals within the bars indicate mean values, while error bars represent standard deviations (SD)

The mean ablation temperature measured at the tip of the electrode after break was 81.0 ± 8.0 °C. The mean ablation temperatures by breast density category: Category A, 76.4 °C (95% CI: 72.8–80.0 °C); Category B, 81.3 °C (95% CI: 77.2–85.4 °C); Category C, 82.7 °C (95% CI: 78.6–86.8 °C); and Category D, 84.0 °C (95% CI: 74.5–93.5 °C). Increased breast densities were associated with high ablation temperatures. Notably, Category C and D groups demonstrated significantly higher temperatures than those noted in Category A group (*p* = 0.037 and *p* = 0.031, respectively) (Fig. 2a). The mean ablation duration until break was 446 ± 139 s. Mean ablation times by breast density category were as follows: Category A, 399 s (95% CI: 295–502 s); Category B, 441 s (95% : 384–499 s); Category C, 470 s (95% CI: 394–567 s); and Category D, 527 s (95% CI: 353–701 s). Although the difference was not statistically significant, the ablation time tended to be prolonged in breasts with higher densities (Fig. 2b). The results of our post-hoc power analysis revealed that although some comparisons yielded large effect sizes (Cohen's d > 0.8), the statistical power was consistently low across all groups for both tip temperature and ablation time.

**Fig. 3:**
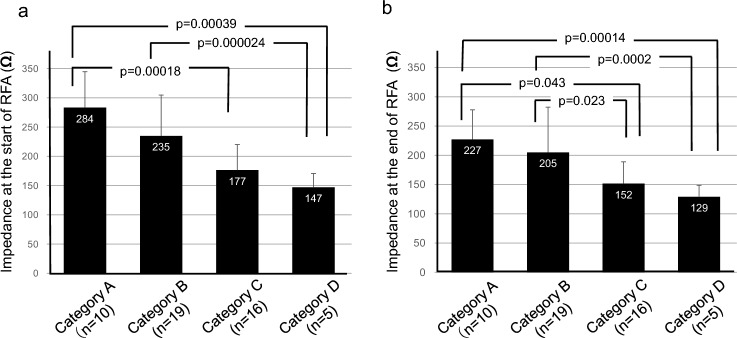
Relationship between breast density and tissue impedance during radiofrequency ablation (RFA). Mean impedance values at the start (**a**) and end (**b**) of RFA for each breast density group. White numerals within the bars represent mean values; meanwhile, error bars indicate standard deviations (SD)

**Fig. 4:**
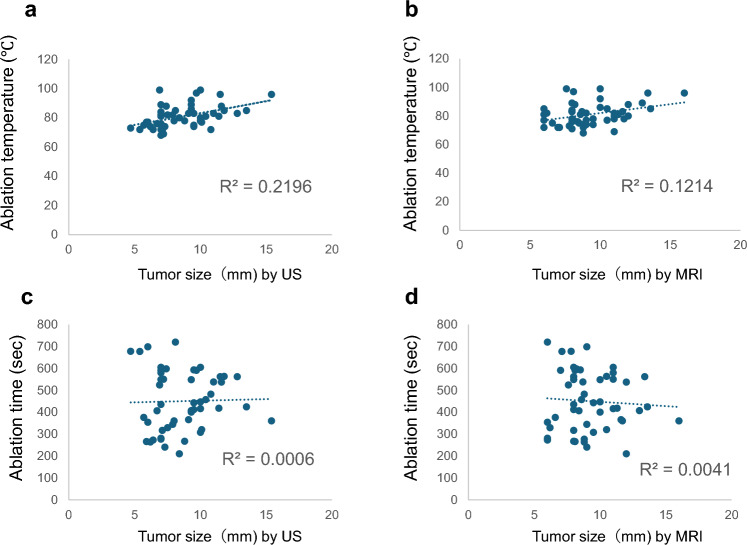
Correlation between tumor size and radiofrequency ablation parameters. The coefficient of determination (R^2^) quantifies the extent to which the tumor diameter, as measured by magnetic resonance imaging (MRI) or ultrasound (US), explains the variance in ablation temperature and duration. **a** Correlation between ablation temperature and tumor size measured using US (R^2^ = 0.2196). **b** Correlation between ablation temperature and tumor size assessed via MRI (R^2^ = 0.1214). **c** Relationship between ablation duration and tumor size measured using US (R^2^ = 0.0006). **d** Correlation between ablation time and tumor size evaluated using MRI (R^2^ = 0.0041)

The impedance values at the start and end of RFA were also analyzed. The mean initial impedance was 208 ± 72.3 Ω. By breast density, the mean initial impedance values were as follows: Category A, 284 Ω (95% CI: 240–327 Ω); Category B, 235 Ω (95% CI: 201–269 Ω); Category C, 177 Ω (95% CI:153–200 Ω); and Category D, 147 Ω (95% CI: 118–176 Ω). High fat content was significantly associated with elevated initial impedance. In particular, Category A and B groups demonstrated significantly higher impedances than those observed in Category D group (*p* = 0.00039 and *p* = 0.00018, respectively) (Fig. 3a). The mean final impedance was 161 ± 66.5 Ω. Additionally, the final impedance by breast density category were as follows: Category A, 227 Ω (95% CI: 191–263 Ω); Category B, 205 Ω (95% CI: 168–242 Ω); Category C, 152 Ω (95% CI:132–171 Ω); and Category D, 129 Ω (95% CI: 104–153 Ω). Final impedance values were significantly higher in breasts with greater fat content. Categories A and B also revealed significantly higher impedance values than those observed in Categories C and D (*p* = 0.00014 and *p* = 0.0002 vs. Category D, and *p* = 0.043 and *p* = 0.023 vs. Category C, respectively) (Fig. 3b). Finally, we examined the relationship between tumor size (as measured using US and MRI) and ablation parameters. Neither the peak ablation temperature nor the ablation time exhibited a strong correlation with tumor size (Fig. 4).

## Discussion

RFA received insurance coverage approval in Japan in 2023 and is anticipated to become a leading minimally invasive local treatment for breast cancer. However, the current evidence remains limited, and further investigations from various perspectives are warranted. Both patient-related and device-specific factors, including those associated with the cool-tip system, are likely to influence efficiency, effectiveness, and safety. During our experience with RFA for early-stage breast cancer, we noticed variability in ablation temperatures and durations across cases. This observation led us to investigate the relationship between breast density and RFA parameters.

Before the clinical application of RFA, several experimental studies examined the thermal effects of the modality in vivo. RFA for breast cancer differs substantially from its application in other solid tumors, such as those of the liver, kidney, and bone. A key distinction lies in the high adipose content of the breast tissue, which markedly alters both electrical and thermal conductivity. Furthermore, RFA for breast cancer is performed with curative intent, particularly in cases of early-stage disease [23]. Boehm et al. performed RFA on rabbit mammary tumors implanted in fatty tissue and suggested that variations in the thermal and electrical conductivities of mammary tissues influenced heat propagation during RFA [28]. In uniform animal tumor models, Ahmed et al. demonstrated that the presence of blood flow reduced the ablation area, indicating that the vascularity and conductivity of surrounding tissues may influence RFA outcomes [29]. Additional complexities may arise in breast tissue, which contains a high proportion of fat. Adipose tissue typically exhibits low thermal and electrical conductivities, potentially causing heat to concentrate within the tumor while limiting its propagation to the surrounding tissue. Therefore, although the tumor core may be adequately ablated, the heat distribution near the tumor margins may be uneven in fatty breast tissues [30, 31].

The potential effect of a 5% glucose injection should also be considered. To prevent thermal injury during RFA, approximately 10–20 mL of a 5% glucose solution was injected in both s the retromammary space and subcutaneous tissue before energy delivery. Unlike saline, a glucose solution does not promote unintended electrical conduction and rarely disperses extensively into adjacent tissues, including those surrounding the tumor. Therefore, the solution is considered to exert a minimal influence, allowing for a relatively controlled ablation of the tumor [32]. The cool-tip system features ablation tips with active lengths of 2 cm or 3 cm. Complete ablation of the tumor core can generally be achieved when the tumor size falls within the active length range of the ablation tip. Previous studies have consistently reported that tumors measuring ≤ 2 cm in diameter can be effectively ablated. The 1.5 cm tumor size criterion adopted in the RAFAELO trial is therefore considered appropriate for safe and effective RFA [24]. Representative cases illustrating the relationship between breast density and the extent of ablation are displayed in Supplementary Fig. S1. MRI and US images obtained approximately three months after the completion of radiation therapy following RFA are presented. In cases classified as Category A, with high fat content, the ablation zones were generally limited, often measuring less than 30 mm. In contrast, among patients with high breast density, especially Categories C and D, ablation areas exceeding 30 mm were observed more frequently (Supplementary Fig. S1). In the present study, two patients exhibited relatively low ablation temperatures (68 °C and 69 °C), both of whom had breast densities classified as Category A or B (Supplementary Fig. S2). According to the eligibility criteria for RFA, these two cases should have been converted to partial mastectomy due to the potential risk of insufficient ablation. However, based on the patient’s preference, close observation was performed in lieu of surgical resection. The ablation zones assessed using MRI and US were approximately 20 mm in diameter and appeared slightly smaller than those in other cases. In the presence of unexpected ductal extension, insufficient ablation may occur, especially in fatty breast tissue, where the thermal conductivity tends to be low and the ablation area is narrow. Fortunately, no viable tumor cells were detected in tissue biopsies performed three months after radiation therapy in either case; however, ongoing close follow-up remains essential.

Although we observed a correlation between breast density and peak ablation temperature in this study, abrupt temperature drops were not detected 1 cm above the ablation site, indicating adequate ablation of the tumor interior. Breast density is influenced by a wide range of factors, including age, hormonal status, BMI, ethnicity, reproductive and breastfeeding history, and lifestyle habits. These factors are also associated with breast cancer risk [33]. Although the present study did not analyze these background factors, future research should consider incorporating such variables to better evaluate the relationship between breast density and RFA. In this study, the BI-RADS classification, a qualitative and visual assessment, was used by independent radiologists as an indicator of the proportion of fat and fibroglandular tissue, which may lack objectivity [27]. Future research may require a novel quantitative assessment method, such as Volpara Health, which offers a clinically validated breast density assessment to enhance accuracy [34]. In clinical practice, ablation is monitored in real time using US to assess treatment progress. The reduced RFA temperatures observed in fatty breasts may be attributed to elevated tissue impedance and shorter ablation time, as the cool-tip needle, positioned within the fat-rich tissue, leads to earlier termination of energy delivery (break) owing to higher baseline impedance. We believe that incorporating breast density into treatment planning may enable a more accurate estimation of the required ablation time and temperature, thereby assisting in the evaluation of the risk of incomplete ablation. In cases of intraductal spreading, fatty breast tissue may exhibit lower thermal conductivity, leading to a reduced ablation zone and increased risk of insufficient ablation. According to the manufacturer, the cool-tip system triggers a break when the impedance increases by 30 Ω or 30% above the baseline value, whichever is greater. Thus, in fatty breast tissue, breaks may occur earlier due to the inherently higher impedance, resulting in reduced energy delivery and, consequently, lower peak temperatures. As illustrated in Fig. 4, the data demonstrated no clear correlation between tumor size and ablation temperature or duration, suggesting that adherence to appropriate patient selection criteria (i.e., tumor size ≤ 1.5 cm) can yield consistently favorable RFA outcomes regardless of background mammary tissue composition.

To the best of our knowledge, this is the first study to report the clinical relationship between breast density and RFA parameters in patients with early-stage breast cancer. Paruch employed an electroheating model that coupled electric and thermal fields to mathematically simulate RFA in breast tissues. Using the estimated temperature distributions, the extent of tissue destruction was measured, and the differences in the ablation effects associated with the breast density composition were analyzed. In fatty breast tissue, characterized by thermal conductivity, the temperature rise tends to be localized, whereas in extremely dense breast tissue, heat disperses more broadly, leading to a more uniform temperature elevation throughout the tumor [35]. The results of the present study are consistent with the values predicted by the electroheating model.

However, this study had several limitations. First, the primary limitation of this study was its single-center design and small sample size, which may limit the generalizability of our findings to other populations. In particular, the limited sample rendered the study underpowered. Although our post-hoc power analysis indicated that some observed differences were of practical significance, they did not achieve statistical significance. Therefore, a large-scale multicenter study is warranted to validate these promising results. An a priori power analysis should be integral to the study design to ensure an adequate sample size. Second, breast density may not directly reflect the fat content of the tissue surrounding the tumor, which may vary across different regions of the breast. In this study, BI-RADS classification was used as an indicator of the proportion of fat and fibroglandular tissue evaluated by three independent radiologists. Future studies may benefit from incorporating additional quantitative assessment methods. Third, we did not consider the potential influence of tissue perfusion (blood flow) on the ablation outcomes. To minimize the impact of blood flow, Doppler US was employed to identify the vascular structures, and the puncture route was carefully selected to avoid them. However, the blood flow within the ablation zone and the injected 5% glucose solution may affect the ablation process. Fourth, RFA outcomes may be affected by various factors, including tumor location, skin-to-tumor distance, amount of surrounding adipose tissue, cool-tip selection, and operator-dependent technical variations. We believe that the standardization of RFA should continue to be advanced under the guidance of the RFA Working Group of the Japanese Breast Cancer Society [36]. Fifth, currently, no well-established method is available for evaluating ablation zones through histological assessment performed three months after post-treatment radiotherapy. Moreover, the long-term outcomes, including local recurrence rates and cosmetic results, remain uncertain. Continued accumulation of clinical data is essential to establish stronger evidence and further develop RFA as a treatment modality originating in Japan.

In conclusion, we investigated the relationship between RFA and breast density in patients with early-stage breast cancer. In fatty breast tissue, the ablation impedance was higher, the peak temperatures were lower, and the ablation time tended to be shorter. We believe that considering breast density may allow for an approximate estimation of the required ablation time and temperature, thereby helping to assess the risk of incomplete ablation. These findings suggest that RFA should be performed with careful consideration of breast density, as fatty breast tissue may result in a narrow ablation zone.

## Supplementary Information

Below is the link to the electronic supplementary material.

Supplementary file1 (PPTX 509 KB)** Fig. S1** Representative cases displaying the relationship between breast density and the extent of ablation. Magnetic resonance imaging (MRI) and ultrasound (US) images obtained approximately three months after the completion of radiation therapy following radiofrequency ablation are displayed. From low-density (Category A) to high-density breast cases (Category D), yellow arrows indicate the approximate ablation sizes on US images (axial view: left, sagittal view: right) measuring 27 × 15 mm, 24 × 18 mm, 27 × 17 mm, and 31 × 22 mm, respectively. Corresponding measurements on MRI (axial view) are 21 × 18 mm, 26 × 21 mm, 33 × 26 mm, and 35 × 27 mm, indicating a tendency for larger ablation zones in denser breast categories.**Fig. S2** Ablation zones in two cases where the ablation temperature did not reach 70 °C. Magnetic resonance imaging (MRI) (axial view) and ultrasound (US) (axial view: left, sagittal view: right) images obtained approximately three months after the completion of radiation therapy following radiofrequency ablation are displayed for two cases. The yellow arrows indicate the ablation zones. Case 1: A 68-year-old female with Category A breast density had an approximate ablation size of 19 mm × 15 mm on US and 18 mm × 17 mm on MRI. Case 2: A 42-year-old female with Category B breast density had an ablation size measuring 19 mm × 16 mm on US and 25 mm × 18 mm on MRI.
