# Supplementary material for: Impact of breast density on the efficacy of radiofrequency ablation in early-stage breast cancer
Source: Breast Cancer. 2025 Sep 22;33(1):72–9. doi: 10.1007/s12282-025-01775-7 (PMC12789161; doi:10.1007/s12282-025-01775-7)
Supplement: Supplementary file 1 — Supplementary file1 (PPTX 509 KB) Fig. S1 Representative cases displaying the relationship between breast density and the extent of ablation. Magnetic resonance imaging (MRI) and ultrasound (US) images obtained approximately three months after the completion of radiation therapy following radiofrequency ablation are displayed. From low-density (Category A) to high-density breast cases (Category D), yellow arrows indicate the approximate ablation sizes on US images (axial view: left, sagittal view: right) measuring 27 × 15 mm, 24 × 18 mm, 27 × 17 mm, and 31 × 22 mm, respectively. Corresponding measurements on MRI (axial view) are 21 × 18 mm, 26 × 21 mm, 33 × 26 mm, and 35 × 27 mm, indicating a tendency for larger ablation zones in denser breast categories.Fig. S2 Ablation zones in two cases where the ablation temperature did not reach 70 °C. Magnetic resonance imaging (MRI) (axial view) and ultrasound (US) (axial view: left, sagittal view: right) images obtained approximately three months after the completion of radiation therapy following radiofrequency ablation are displayed for two cases. The yellow arrows indicate the ablation zones. Case 1: A 68-year-old female with Category A breast density had an approximate ablation size of 19 mm × 15 mm on US and 18 mm × 17 mm on MRI. Case 2: A 42-year-old female with Category B breast density had an ablation size measuring 19 mm × 16 mm on US and 25 mm × 18 mm on MRI. [file 12282_2025_1775_MOESM1_ESM.pptx]

## Slide 1
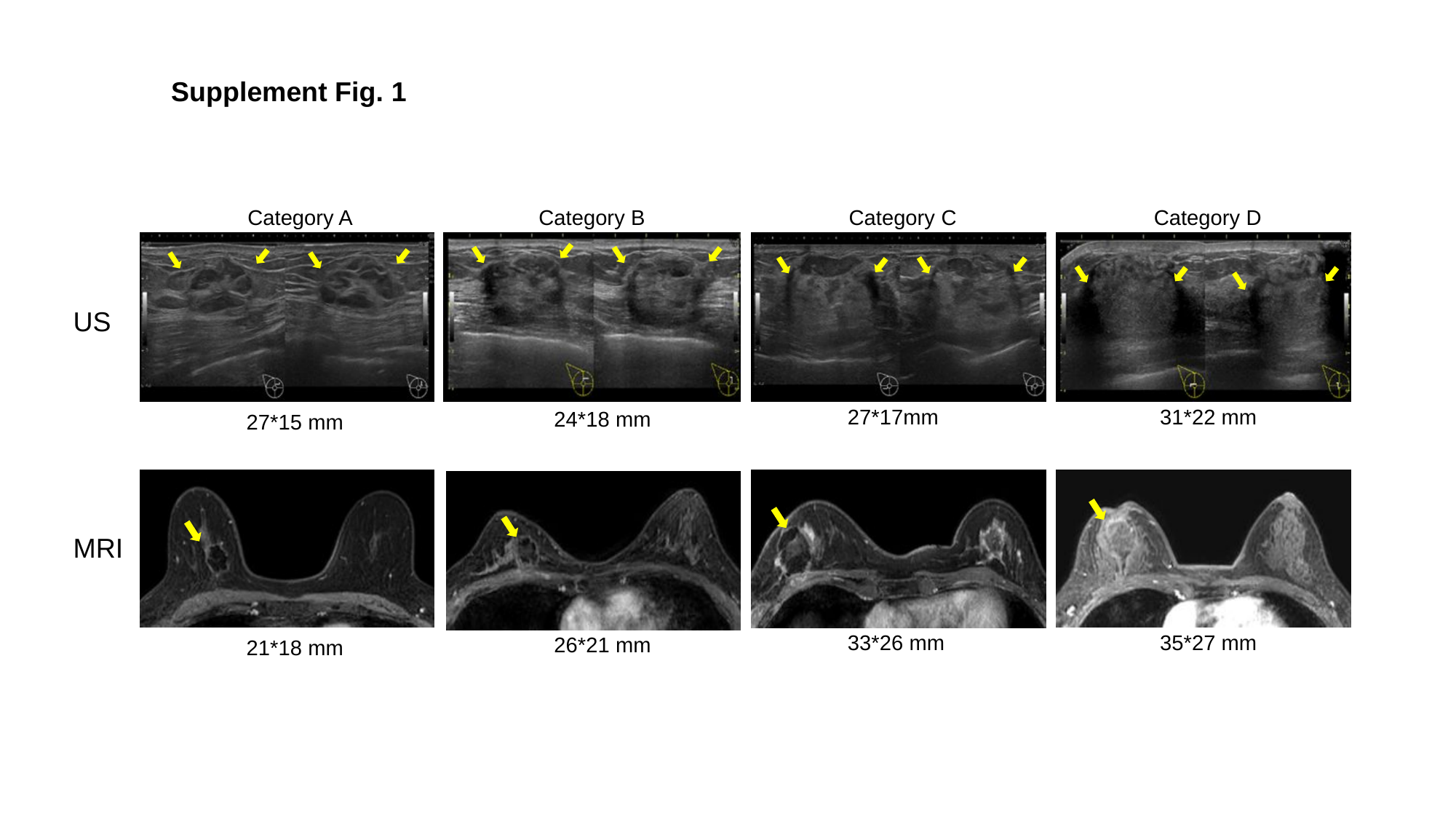

Supplement Fig. 1
Category A
Category B
Category C
Category D
US
27*17mm
31*22 mm
24*18 mm
27*15 mm
MRI
33*26 mm
35*27 mm
26*21 mm
21*18 mm

## Slide 2
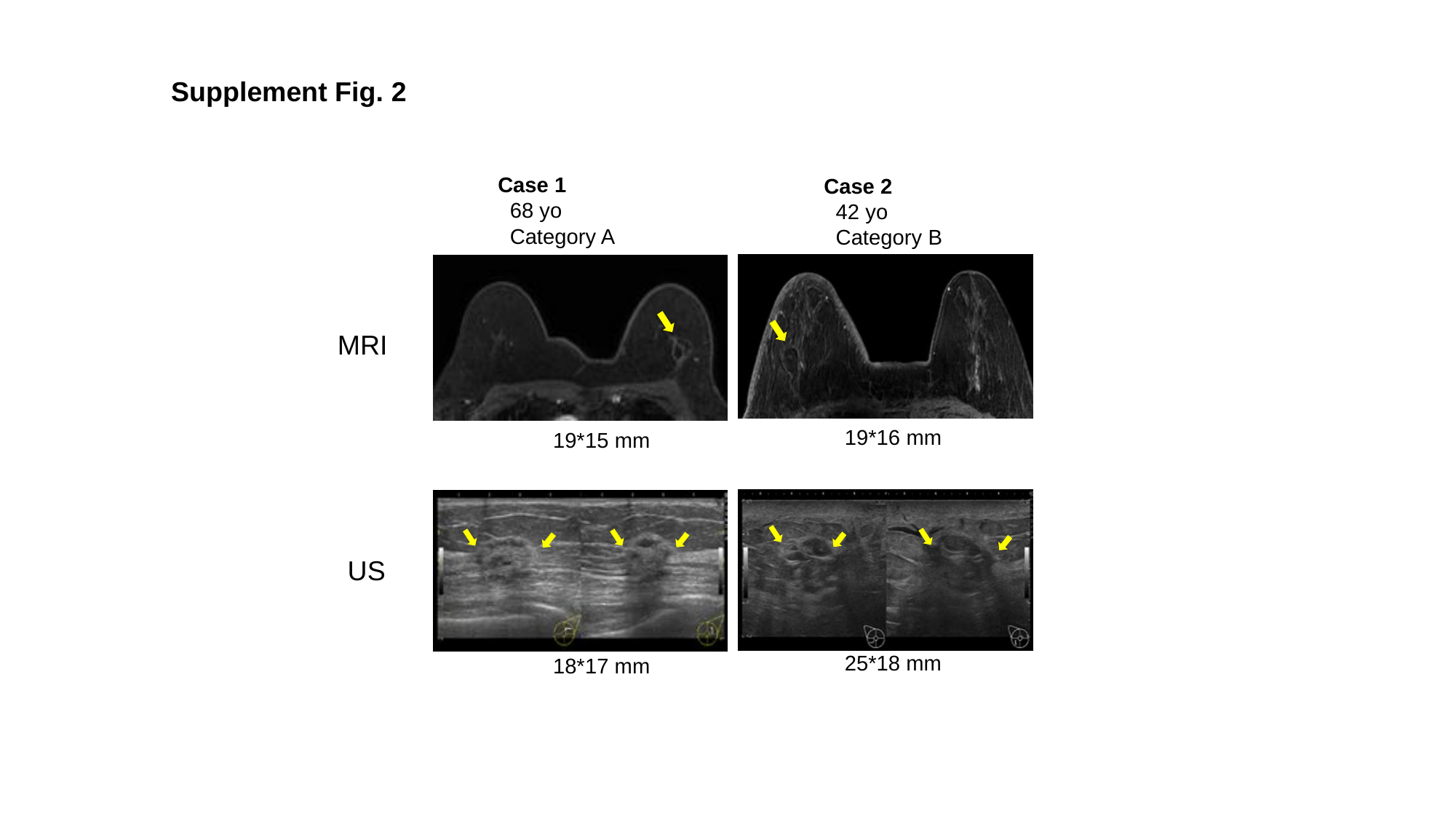

Supplement Fig. 2
Case 1
 68 yo
 Category A
Case 2
 42 yo
 Category B
MRI
19*16 mm
19*15 mm
US
25*18 mm
18*17 mm
